# The influencing factors of postoperative sleep disorders in elderly patients undergoing hip replacement surgery under general anesthesia and the construction and validation of a nomogram prediction model

**DOI:** 10.3389/fneur.2025.1655523

**Published:** 2025-11-27

**Authors:** Guo Zhou, Jun Chen, Hui Zhang

**Affiliations:** 1 Department of Surgical Anesthesiology, Changsha Hospital of Traditional Chinese Medicine, Changsha, Hunan Province, China; 2 Department of Surgical Anesthesiology, Hunan Chest Hospital, Changsha, Hunan Province, China

**Keywords:** general anesthesia, hip replacement surgery, sleep disorders, influencing factors, column chart nomogram

## Abstract

**Objective:**

To explore the influencing factors of postoperative sleep disorders in elderly patients undergoing hip replacement (HR) under general anesthesia, and to construct and validate a nomogram prediction model.

**Methods:**

A total of 329 patients who underwent general anesthesia HR surgery in our hospital from December 2021 to December 2023 were retrospectively gathered and grouped into a modeling group (230 cases) and a validation group (99 cases). The modeling group was separated into a sleep disorder group and a no sleep disorder group based on postoperative sleep disorders.

**Results:**

Out of 230 patients, 69 experienced sleep disorders, with an incidence rate of 30.00%. Multivariate logistic regression found that age, anesthesia time, surgery time, intraoperative blood loss, postoperative hypoxemia, postoperative VAS score, and postoperative CRP level were risk factors for postoperative sleep disorders in elderly HR patients undergoing general anesthesia (*p* < 0.05). The AUC of the modeling group and validation group was 0.978 and 0.972, and the H-L test showed χ^2^ = 7.410 and 7.342, respectively, *p* = 0.762 and 0.752, indicating good consistency. DCA curve showed that when the high-risk threshold probability was between 0.08 and 0.88, the nomogram model had high clinical value.

**Conclusion:**

Age, anesthesia time, surgery time, intraoperative blood loss, postoperative hypoxemia, postoperative VAS score, and postoperative CRP level are the influencing factors of postoperative sleep disorders in elderly HR patients undergoing general anesthesia. The nomogram model constructed based on this has good discrimination and consistency, and can predict the postoperative sleep disorders of patients.

## 1 Introduction

Accompanying the trend of population aging, elderly hip joint diseases have become a clinical concern, with an evident increase in the risk of hip fractures, osteoarthritis, and other hip joint diseases among the elderly population (1). Hip replacement (HR) surgery is a primary clinical treatment method for hip joint diseases, which can reconstruct the patient’s hip joint function, control the progression of the disease, and improve the patient’s quality of life (2). However, the elderly patients’ various physiological functions are relatively low, leading to poor tolerance to surgery and anesthesia, increasing the likelihood of complications. Postoperative sleep disorders are one such complication, mainly referring to a decline in sleep quality and alteration in sleep structure after surgery. Patients may experience sleep disorders due to environmental factors, pain, stress, and other factors during surgery, and postoperative sleep disorders are related to surgical delirium, sympathetic nervous excitement, etc., affecting the patient’s postoperative recovery and surgical efficacy (3, 4). Therefore, it is particularly important in clinical practice to identify the factors influencing postoperative sleep disorders in HR surgery patients to improve their quality of life. Nomograms are simple to operate and highly readable, integrating risk factors screened from regression analysis to accurately predict the risk at a specific time on an individual basis (5, 6). Based on this, there are few reports on the study of postoperative sleep disorders in HR surgery patients using nomograms (7). Therefore, this study aims to explore the influencing factors of postoperative sleep disorders in elderly patients undergoing general anesthesia HR surgery and the construction and validation of a nomogram prediction model.

## 2 Materials and methods

### 2.1 General data

**Figure 1:**
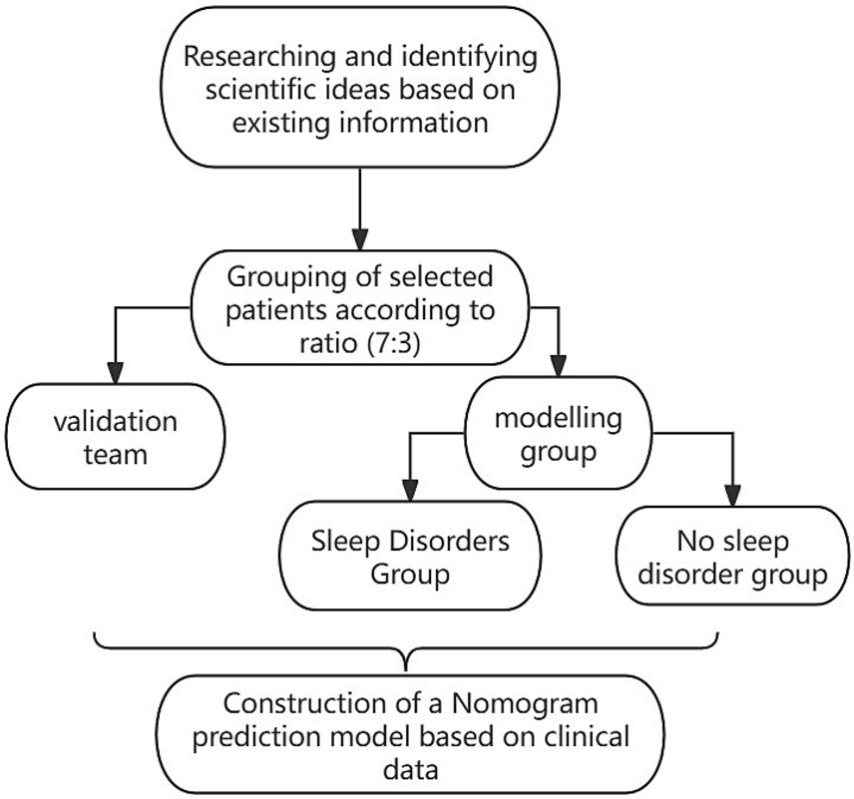
Flow chart of case collection.

A retrospective selection of 329 patients undergoing general anesthesia HR surgery in our hospital from December 2021 to December 2023 was made. Patients were randomly divided into modeling group (230 cases) and validation group (99 cases) in a 7:3 ratio (The random number table method was used, with a random number sequence generated by SPSS software to ensure that the sequence was pattern-free and conformed to the characteristics of a random distribution. The numbers in the random number table were coded from 0 to 9, with 0–6 assigned to the modeling group and 7–9 assigned to the validation group, ensuring a 7:3 allocation ratio). Patients in the modeling group were further divided into sleep disorder group and non-sleep disorder group based on postoperative sleep disorder conditions. The case collection flow chart is shown in Figure 1 (Clinical data of the patients were collected, and the patients were divided into a modeling group and a validation group. The patients in the modeling group were further divided into those with and without sleep disorders to analyze the factors influencing sleep disturbances). Inclusion criteria: (1) meeting the HR treatment indications (8); (2) undergoing general anesthesia; (3) age >60 years; (4) complete data. Exclusion criteria: (1) liver and kidney dysfunction; (2) systemic infectious diseases; (3) autoimmune diseases; (4) malignant tumors; (5) hematological diseases; (6) mental disorders. This study was approved by the hospital ethics committee.

### 2.2 Postoperative sleep disorder assessment

Postoperative sleep disorders were assessed using the Self-Rating Sleep Scale (SRSS) (9), which evaluates whether patients have sleep disorders. It includes 10 items, each scored from 1 to 5, with a total score of 50. A score of <10 indicates no significant sleep disorder, while ≥10 indicates the presence of a sleep disorder.

### 2.3 Clinical data

Clinical data were collected from routine examinations and electronic medical record queries, including age, gender, body mass index (BMI), American Society of Anesthesiologists (ASA) classification, hypertension, diabetes, hyperlipidemia, coronary heart disease, atrial fibrillation, cerebral infarction, smoking history, alcohol history, disease type, cognitive impairment, depression, nutritional disorder, anxiety, anesthesia time, surgery time, preoperative bleeding volume, surgical method, postoperative hypoxemia, postoperative pain Visual Analog Scale (VAS)(assessed at 24 h after surgery), and postoperative C-reactive protein (CRP) levels(measured at 24 h after surgery).

### 2.4 Statistical processing

Data were analyzed using SPSS 25.0. Categorical data were tested using the χ^2^ test, expressed as cases (%). Continuous data were tested using the t-test, expressed as $\bar{x}\pm s$ . Influencing factors of postoperative sleep disorders in elderly patients undergoing general anesthesia HR surgery were analyzed using multivariate logistic regression. The nomogram model for postoperative sleep disorders in elderly patients undergoing general anesthesia HR surgery was constructed using R software. The ROC curve was drawn to evaluate the discrimination of the nomogram model for postoperative sleep disorders in elderly patients undergoing general anesthesia HR surgery; the calibration curve was drawn to evaluate the model consistency; the clinical decision curve (DCA) was used to evaluate the clinical application value of the model. *p* < 0.05 was considered significant.

## 3 Results

### 3.1 Comparison of clinical data between modeling group and validation group

There were no significant differences in clinical data between the modeling group and the validation group (*p* > 0.05). See Table 1.

**Table 1:** Comparison of clinical data between modeling and validation group.

Considerations	Modeling group (*n* = 230)	Validation group (*n* = 99)	*t/χ* ^2^	*p*
Age (years)			0.176	0.675
≥70	101(43.91)	41(41.41)		
<70	129(56.09)	58(58.59)		
Genders			0.129	0.720
Man	135(58.70)	56(56.57)		
Woman	95(41.30)	43(43.43)		
BMI(kg/m^2^)			0.168	0.681
<23	138(60.00)	57(57.58)		
≥23	92(40.00)	42(42.42)		
ASA classification			0.554	0.457
Level II	49(21.30)	24(24.24)		
Level III	177(76.96)	72(72.73)		
Level IV	4(1.74)	3(3.03)		
Hypertensive			0.169	0.681
Yes	75(32.61)	30(30.30)		
No	155(67.39)	69(69.70)		
Diabetes			0.177	0.674
Yes	68(29.57)	27(27.27)		
No	162(70.43)	72(72.73)		
Hypertriglyceridemia			0.035	0.851
Yes	58(25.22)	24(24.24)		
No	172(74.78)	75(75.76)		
Coronary heart disease			0.351	0.554
Yes	41(17.83)	15(15.15)		
No	189(82.17)	84(84.85)		
Atrial fibrillation			0.011	0.918
Yes	17(7.39)	7(7.07)		
No	213(92.61)	92(92.93)		
Cerebral infarction			0.089	0.765
Yes	16(6.96)	6(6.06)		
No	214(93.04)	93(93.94)		
Smoking history			0.036	0.851
Yes	58(25.22)	24(24.24)		
No	172(74.78)	75(75.76)		
Drinking history			0.110	0.740
Yes	55(23.91)	22(22.22)		
No	175(76.09)	77(77.78)		
Type of disease			2.091	0.148
Aseptic necrosis of the femoral head	30(13.04)	20(20.20)		
Intertrochanteric fracture of the femur	55(23.91)	25(25.25)		
Femoral neck fracture	145(63.04)	54(54.55)		
With cognitive impairment			0.138	0.711
Yes	105(45.65)	43(43.43)		
No	125(54.35)	56(56.57)		
With depression			0.073	0.787
Yes	59(26.65)	24(24.24)		
No	171(74.35)	75(75.76)		
With nutritional disorders			0.047	0.828
Yes	56(24.35)	23(23.23)		
No	174(75.65)	76(76.77)		
With anxiety			0.098	0.755
Yes	50(21.74)	20(20.20)		
No	180(78.26)	79(79.80)		
Anesthesia time(h)			0.138	0.710
≥2	112(48.70)	46(46.46)		
<2	118(51.30)	53(53.54)		
Surgical time(min)			0.075	0.784
≥120	106(46.09)	44(44.44)		
<120	124(53.91)	55(55.56)		
Intraoperative hemorrhage(mL)			0.104	0.747
≥300	109(47.39)	45(45.45)		
<300	121(52.61)	54(54.55)		
Surgical procedure			0.250	0.617
Total hip replacement	130(56.52)	53(53.54)		
Hemiarthroplasty	100(43.48)	46(46.46)		
Postoperative hypoxemia			0.031	0.859
Yes	100(43.48)	42(42.42)		
No	130(56.52)	57(57.58)		
Postoperative VAS score(points)			0.138	0.711
>3	105(45.65)	43(43.43)		
≤3	125(54.35)	56(56.57)		
Postoperative CRP levels(mg/L)	55.09 ± 12.17	54.86 ± 12.05	0.158	0.875

### 3.2 Comparison of clinical data between sleep disorder group and non-sleep disorder group

Among the 230 patients, 69 experienced sleep disorders, with an incidence rate of 30.00%. There were significant differences between the two groups in terms of age, anesthesia time, surgery time, intraoperative bleeding volume, postoperative hypoxemia, postoperative VAS score, and postoperative CRP levels (*p* < 0.05). There were no significant differences in other clinical data between the two groups (*p* > 0.05). See Table 2.

**Table 2:** Comparison of clinical data between the sleep disordered and non-sleep disordered groups.

Considerations	Sleep disorders group (*n* = 69)	No sleep disorder group (*n* = 161)	*t/χ* ^2^	*p*
Age (years)			11.507	0.001
≥70	42(60.87)	59(36.65)		
<70	27(39.13)	102(63.35)		
Genders			0.021	0.884
Man	40(57.97)	95(59.01)		
Woman	29(42.03)	66(40.99)		
BMI(kg/m^2^)			0.221	0.638
<23	43(62.32)	95(59.01)		
≥23	26(37.68)	66(40.99)		
ASA classification			0.775	0.379
Level II	15(21.74)	34(21.12)		
Level III	52(75.36)	125(77.64)		
Level IV	2(2.90)	2(1.24)		
Hypertensive			0.212	0.645
Yes	21(30.43)	54(33.54)		
No	48(69.57)	107(66.46)		
Diabetes			0.573	0.449
Yes	18(26.09)	50(31.06)		
No	51(73.91)	111(68.94)		
Hypertriglyceridemia			0.215	0.643
Yes	16(23.19)	42(26.09)		
No	53(76.81)	119(73.91)		
Coronary heart disease			0.748	0.387
Yes	10(14.49)	31(19.25)		
No	59(85.51)	130(80.75)		
Atrial fibrillation			0.003	0.956
Yes	5(7.25)	12(7.45)		
No	64(92.75)	149(92.55)		
Cerebral infarction			0.013	0.910
Yes	5(7.25)	11(6.83)		
No	64(92.75)	150(93.17)		
Smoking history			0.215	0.643
Yes	16(23.19)	42(26.09)		
No	53(76.81)	119(73.91)		
Drinking history			0.256	0.613
Yes	15(21.74)	40(24.84)		
No	54(78.26)	121(75.16)		
Type of disease			2.905	0.088
Aseptic necrosis of the femoral head	10(14.49)	20(12.42)		
Intertrochanteric fracture of the femur	21(30.43)	34(21.12)		
Femoral neck fracture	38(55.07)	107(66.46)		
With cognitive impairment			0.021	0.885
Yes	31(44.93)	74(45.96)		
No	38(55.07)	87(54.04)		
With depression			0.053	0.818
Yes	17(24.64)	42(26.09)		
No	52(75.36)	119(73.91)		
With nutritional disorders			0.072	0.789
Yes	16(23.19)	40(24.84)		
No	53(76.81)	121(75.16)		
With anxiety			0.122	0.727
Yes	14(20.29)	36(22.36)		
No	55(79.71)	125(77.64)		
Anesthesia time(h)			14.881	<0.001
≥2	47(68.12)	65(40.37)		
<2	22(31.88)	96(59.63)		
Surgical time(min)			14.519	<0.001
≥120	45(65.22)	61(37.89)		
<120	24(34.78)	100(62.11)		
Intraoperative hemorrhage(mL)			7.182	0.007
≥300	42(60.87)	67(41.61)		
<300	27(39.13)	94(58.39)		
Surgical procedure			0.084	0.772
Total hip replacement	40(57.97)	90(55.90)		
Hemiarthroplasty	29(42.03)	71(44.10)		
Postoperative hypoxemia			14.238	<0.001
Yes	43(62.32)	57(35.40)		
No	26(37.68)	104(64.60)		
Postoperative VAS score(points)			11.036	<0.001
>3	43(62.32)	62(38.51)		
≤3	26(37.68)	99(61.49)		
Postoperative CRP levels(mg/L)	58.42 ± 12.45	47.35 ± 11.52	6.517	<0.001

### 3.3 Analysis of factors influencing postoperative sleep disorders in elderly patients undergoing general anesthesia HR surgery

Using whether patients with HICH had poor prognosis after burr hole drainage as the dependent variable (yes = 1, no = 0), and the aforementioned factors with differences as independent variables, the variable assignment method is shown in Table 3. Multivariate Logistic regression analysis results showed that age, anesthesia time, surgery time, intraoperative bleeding volume, postoperative hypoxemia, postoperative VAS score, and postoperative CRP levels were risk factors for postoperative sleep disorders in elderly patients undergoing general anesthesia HR surgery (*p* < 0.05). See Table 4.

**Table 3:** Independent variable assignment methods.

Variable	Assignment method
Age	<70 years = 0, ≥ 70 years = 1
Anesthesia time	≥2 h = 0, <2 h = 1
Surgical time	≥120 min = 0, <120 min = 1
Intraoperative hemorrhage	≥300 mL = 0, <300 mL = 1
Postoperative hypoxemia	no = 0, yes = 1
Postoperative VAS score	>3 points = 0, ≤ 3 points = 1
Postoperative CRP levels	Continuous variable

**Table 4:** Analysis of factors affecting postoperative sleep disorders in elderly patients undergoing HR with general anesthesia.

Variable	*β* value	SE value	Wald χ^2^ value	*p* value	OR value	95%*CI*
Age	1.970	0.639	9.518	0.002	7.168	2.050–25.070
Anesthesia time	1.531	0.616	6.176	0.013	4.624	1.382–15.473
Surgical time	2.154	0.679	10.046	0.002	8.616	2.275–32.636
Intraoperative hemorrhage	2.686	0.663	16.382	<0.001	14.624	3.989–53.607
Postoperative hypoxemia	2.752	0.658	17.504	<0.001	15.678	4.318–56.917
Postoperative VAS score	1.642	0.644	6.495	0.011	5.164	1.461–18.251
Postoperative CRP levels	3.077	0.736	17.501	<0.001	21.704	5.133–91.770
Constant	−8.265	1.737	22.632	<0.001	<0.001	-

### 3.4 Establishment of a nomogram model for postoperative sleep disorders in elderly patients undergoing general anesthesia HR surgery

**Figure 2:**
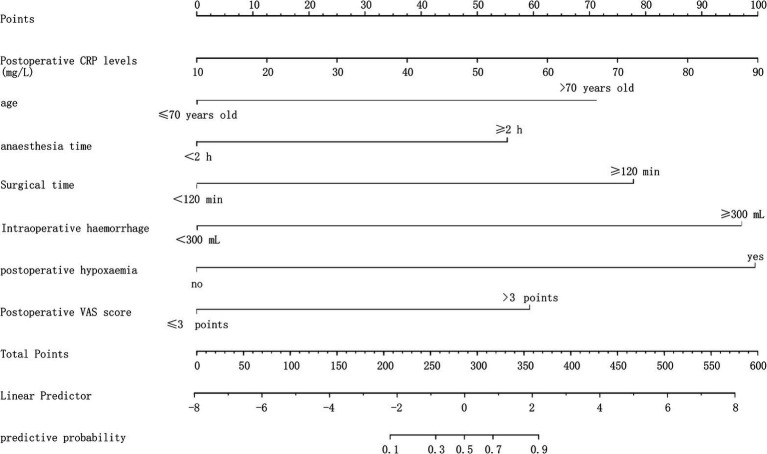
Nomogram modeling of postoperative sleep disorders in elderly patients undergoing general anesthesia for HR.

Based on the factors identified above, a nomogram model was constructed. The predicted probability was calculated as P = e^x / (1 + e^x), where x = −8.265 + 1.970 × age + 1.531 × anesthesia time + 2.154 × surgery time + 2.686 × intraoperative blood loss + 2.752 × postoperative hypoxemia + 1.642 × postoperative VAS score + 3.077 × postoperative CRP level. In this model, the most important influencing factor was postoperative CRP level. For each variable, a vertical line can be drawn to correspond to a specific score. The total score is the sum of the scores for each variable; a higher total score indicates a higher probability of bladder paralysis. For example, at a total score of 320 points, drawing a vertical line to the probability scale corresponds to a predicted probability of 72%. See Figure 2.

### 3.5 Nomogram model for postoperative sleep disorders in elderly patients undergoing general anesthesia HR surgery in the modeling group

**Figure 3:**
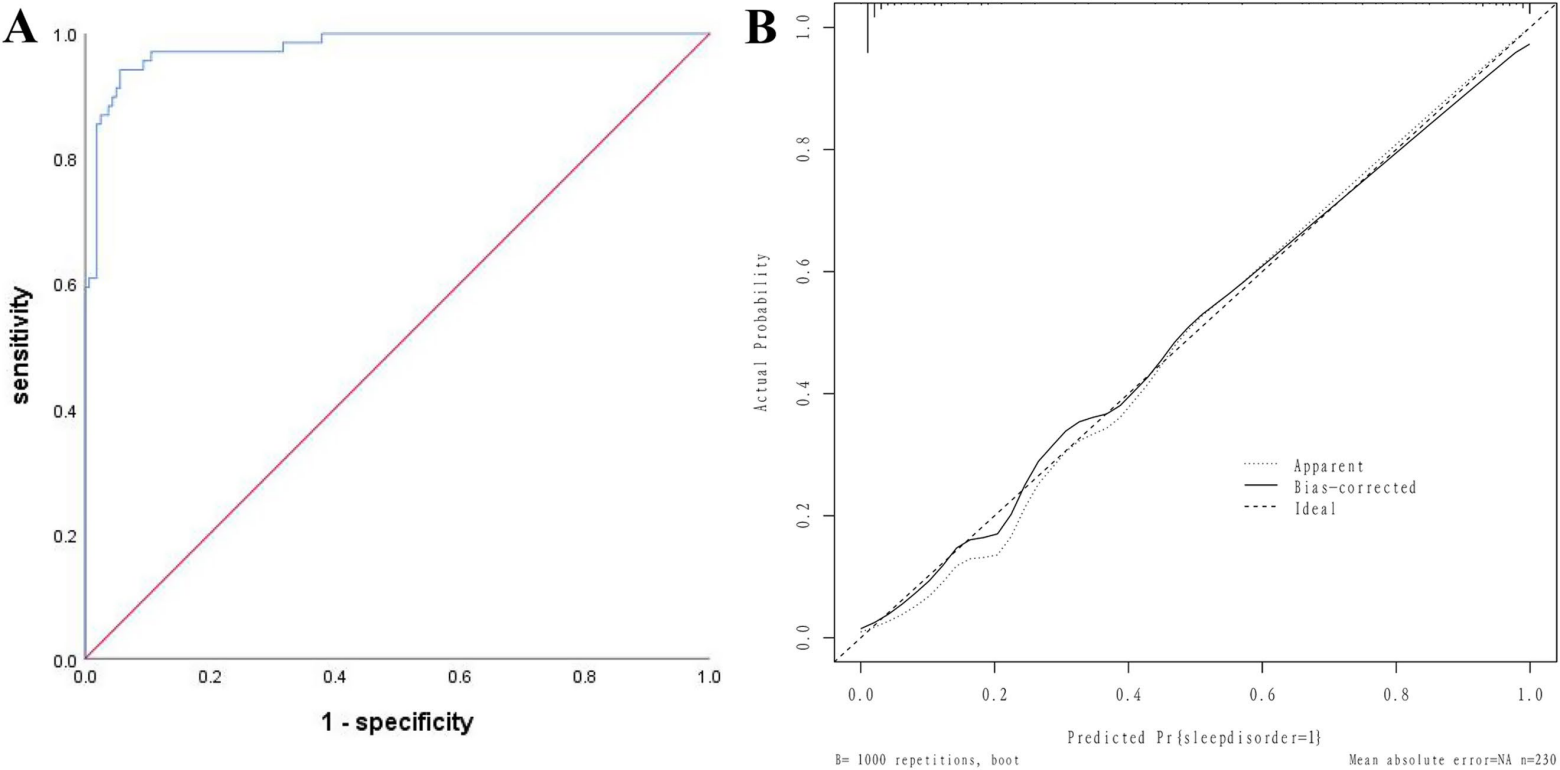
Nomogram Model for postoperative sleep disorders in elderly patients undergoing general anesthesia HR surgery in the modeling group. **(A)** ROC curve for modeling group; **(B)** Modeling group calibration curves.

The AUC of the modeling group was 0.978 (95% CI: 0.962–0.995), the slope of the calibration curve was close to 1, and the H-L test was χ^2^ = 7.410, *p* = 0.762, indicating good consistency. See Figure 3.

### 3.6 Nomogram model for postoperative sleep disorders in elderly patients undergoing general anesthesia HR surgery in the validation group

**Figure 4:**
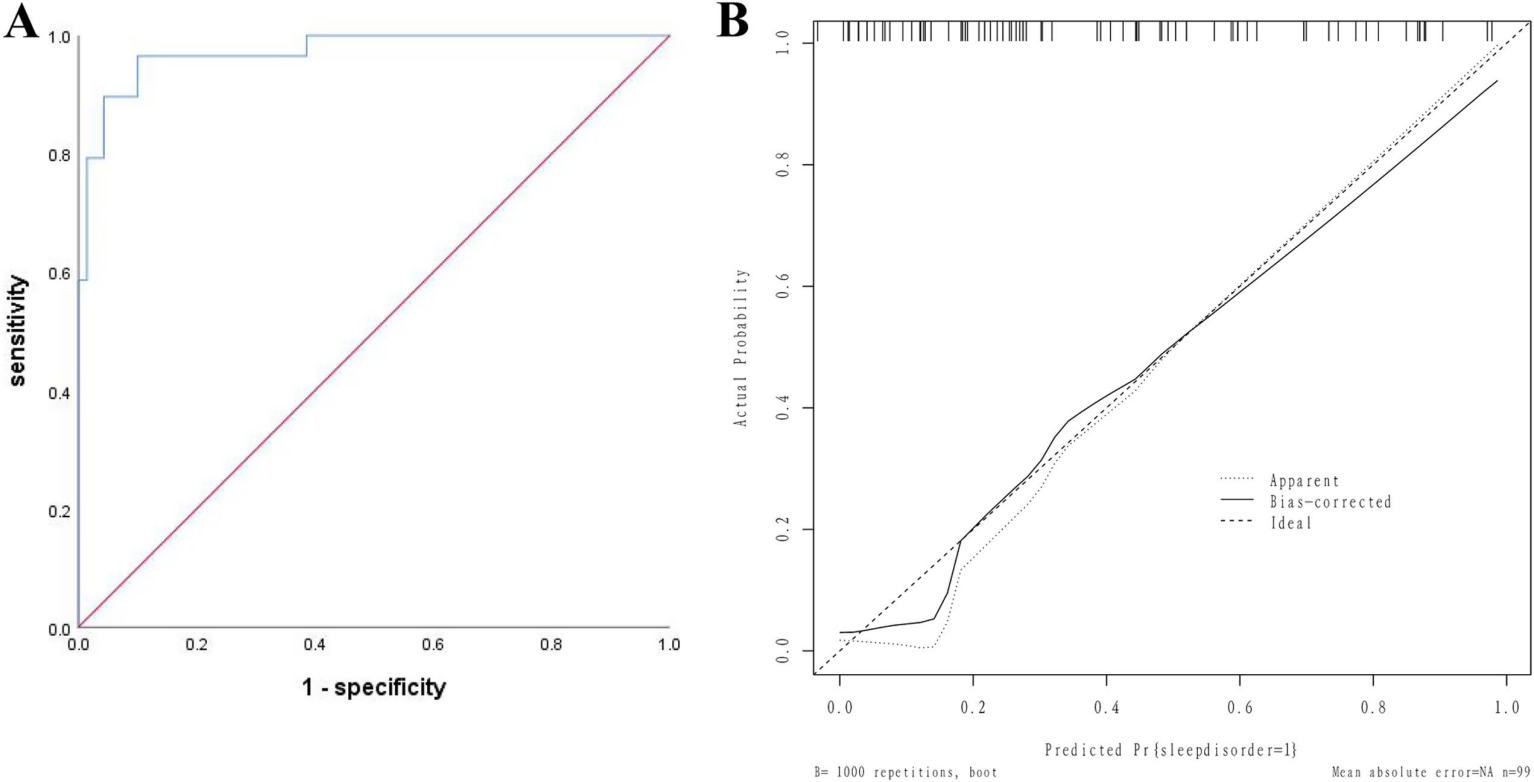
Nomogram model for postoperative sleep disorders in elderly patients undergoing general anesthesia HR surgery in the validation group. **(A)** ROC curve for the validation group; **(B)** Calibration curve for the validation group.

The AUC of the validation group was 0.972 (95% CI: 0.942–0.999); the slope of the calibration curve was close to 1, and the H-L test was χ^2^ = 7.342, *p* = 0.752, indicating good consistency. See Figure 4.

### 3.7 DCA curve of the nomogram model

**Figure 5:**
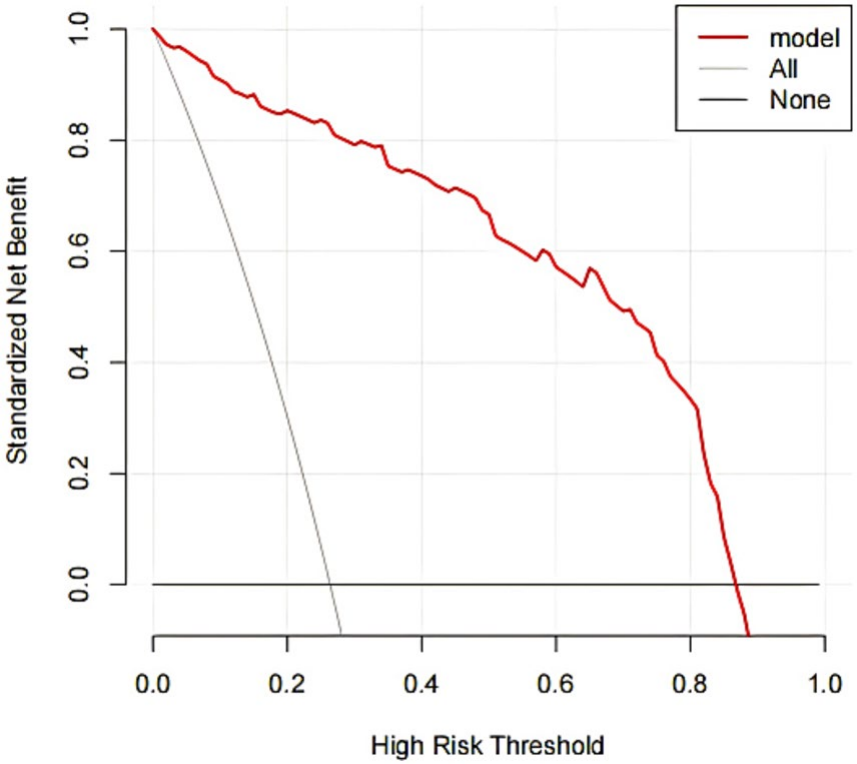
DCA curve for the nomogram.

The DCA curve showed that when the high-risk threshold probability was between 0.08 and 0.88, the clinical use value of using this nomogram model to evaluate postoperative sleep disorders in elderly patients undergoing general anesthesia HR surgery was higher. See Figure 5.

## 4 Discussion

HR is a common clinical treatment method for diseases such as femoral head necrosis and femoral neck fractures, effectively improving patients’ clinical symptoms and quality of life (10). However, due to the stress and pain caused by surgery, patients are prone to postoperative sleep disorders, most of which occur on the first night after surgery. Patients exhibit difficulty falling asleep, shallow sleep, and morning fatigue, which can lead to hyperalgesia and cognitive impairment during the progression, affecting postoperative recovery (11, 12). Therefore, identifying the influencing factors of postoperative sleep disorders and taking measures to treat them can effectively improve patients’ prognosis.

This study screened seven influencing factors and analyzed their causes: (1) For older patients, various physiological functions gradually decline, reducing tolerance to anesthesia and surgery, and the central nervous system functions also degrade to varying degrees. This leads to a decrease in the content of neurotransmitters and receptors in the central system, causing brain energy metabolism disorders, and increasing the risk of postoperative sleep disorders. Studies have found that elderly patients are more likely to experience sleep disturbances after general anesthesia HR surgery, while maintaining a stable internal environment before surgery and removing factors that cause neural injury can effectively reduce the incidence of sleep disorders. (13, 14). (2) Longer anesthesia time also increases sleep disorders, as prolonged general anesthesia alters the patient’s hemodynamics, making them prone to hypotension, resulting in ischemia and hypoxia of brain tissues, affecting brain function and leading to postoperative sleep disorders. In addition, due to individual differences, not all patients can receive the same anesthetic drugs, and the duration of anesthetic administration can significantly increase the total body uptake of anesthesia drugs, which contributes to the increased risk of sleep disorders with prolonged general anesthesia (15). (3) The longer the surgery time, the more likely the patient is to experience intraoperative hypotension and hypoxemia, causing ischemic and hypoxic damage to brain tissues, increasing the likelihood of postoperative sleep disorders (16). (4) Excessive bleeding during HR surgery can cause blood pressure fluctuations and brain hypoxia. Studies have found that excessive bleeding during HR surgery can induce stress reactions, significantly increasing the expression of local inflammatory factors in blood and brain tissues, triggering neuroinflammation and leading to sleep disorders (17, 18). (5) Postoperative hypoxemia aggravates brain tissue stress, reducing blood oxygen saturation, hindering the tricarboxylic acid cycle, decreasing the synthesis of acetylcholine in the central system, and inducing brain cell hypoxia and edema, thereby affecting central nervous system function and increasing the risk of sleep disorders (19). (6) The stronger the postoperative pain, the more intense the patient’s tension and anxiety, directly affecting sleep quality, causing sleep function disorders, and increasing the risk of sleep disorders (20). (7) When CRP levels rise, it can disrupt the blood–brain barrier, and upon entering the brain, cause inflammatory stimulation to nerve cells, stimulate glial cells in the brain to release inflammatory factors, trigger neurotoxic reactions and increase the risk of postoperative sleep disorders (21). Clinically, for these patients, correcting electrolyte imbalances, improving the internal environment and hypoxemic conditions, and selecting appropriate pharmacological treatments can help reduce the risk of postoperative sleep disorders.

This study constructed a nomogram model for postoperative sleep disorders in elderly patients undergoing general anesthesia HR surgery. The AUC of the modeling group and validation group were 0.978 and 0.972, respectively, indicating high discrimination. The high values may be due to the relatively small sample size, and as this is a retrospective study, there may be bias in data selection. Further validation with an expanded sample size will be conducted. The slope of the calibration curve was close to 1, indicating good consistency between the model’s risk assessment and actual risk. Additionally, the DCA curve showed that when the high-risk threshold probability was between 0.08 and 0.88, the clinical use value of this nomogram model was high, helping clinicians assess the risk of postoperative sleep disorders based on influencing factors and timely prevention.

In addition, several variables that were initially thought to be associated with postoperative sleep disorders, including gender, BMI, ASA classification, hypertension, diabetes, hyperlipidemia, coronary heart disease, atrial fibrillation, cerebral infarction, smoking history, alcohol history, cognitive impairment, depression, nutritional disorder, and anxiety, were not found to be significantly associated with postoperative sleep disorders in our study (*p* > 0.05). This finding highlights that not all commonly considered clinical factors contribute to postoperative sleep disturbances in elderly patients undergoing hip replacement surgery under general anesthesia.

In summary, age, anesthesia time, surgery time, intraoperative bleeding volume, postoperative hypoxemia, postoperative VAS score, and postoperative CRP levels are influencing factors for postoperative sleep disorders in elderly patients undergoing general anesthesia HR surgery. The constructed nomogram model shows good discrimination and consistency, predicting postoperative sleep disorder conditions. This study has several limitations. As a single-center, retrospective study with a relatively small sample size, the results may be subject to bias, and the limited sample size may have prevented the detection of some factors related to postoperative sleep disorders. In addition, due to individual differences in patients’ physical conditions, different anesthetic drugs were used, some of which may be associated with a higher risk of postoperative sleep disturbances. In future studies, we plan to expand the sample size and conduct prospective, multicenter studies to further validate our findings and explore other potential influencing factors.

## Data Availability

The original contributions presented in the study are included in the article/supplementary material, further inquiries can be directed to the corresponding author.
